# EXPLORING THE ASSOCIATION BETWEEN CONTRACT NURSE STAFFING AND NURSE TURNOVER IN NURSING HOMES

**DOI:** 10.1093/geroni/igad104.0749

**Published:** 2023-12-21

**Authors:** Neeraj Dayama, Ganisher Davlyatov, Rohit Pradhan, Robert Weech-Maldonado

**Affiliations:** Texas Tech University Health Sciences Center, Lubbock, Texas, United States; University of Oklahoma Health Sciences Center, Oklahoma City, Oklahoma, United States; Texas State University, San Marcos, Texas, United States; University of Alabama at Birmingham, Birmingham, Alabama, United States

## Abstract

NHs face a significant problem with nurse staffing---a critical factor in providing high-quality care to residents. Although contract nurse staffing in NHs may provide a relatively flexible option to address acute nurse shortages, it raises its own challenges including requirement of increased supervision, lower levels of teamwork, and increased workload for the permanent staff. This may lead to disenchantment of the remaining nursing staff, increased turnover---a perfect vicious cycle. Therefore, we examined whether higher ratios of contract nurse utilization were associated with higher nurse turnover rates (RNs, LPNs, and CNAs). Employing a pooled cross-sectional observational study design, we extracted secondary data from PBJ nurse staffing, Nursing Home Compare, Rural-urban commuting area (RUCA) codes, and American Community Survey (ACS) for the period 2017-2021. We employed multivariate mixed-effects maximum likelihood regression to estimate the relationship between the ratios of contract nurses and nurse turnover rates while controlling for organizational/community variables. We ran three separate regressions for RNs, LPNs, and CNAs. Our results suggest that NHs with a high proportion of contract nurses had higher nurse turnover rates for all the three types of staffing (RNs, LPNs, and CNAs). Moreover, NHs with high RN turnover rates had low RN staffing, higher CNA staffing, lower occupancy rates, and higher share of Medicaid residents. High nurse turnover can adversely affect resident outcomes—for instance, due to reduced continuity of care. As such, it is important to closely monitor contract nurse staffing levels and their implications on staffing and resident outcomes.

